# A Single Session of Anodal Cerebellar Transcranial Direct Current Stimulation Does Not Induce Facilitation of Locomotor Consolidation in Patients With Multiple Sclerosis

**DOI:** 10.3389/fnhum.2020.588671

**Published:** 2020-10-30

**Authors:** Carine Nguemeni, György A. Homola, Luis Nakchbandi, Mirko Pham, Jens Volkmann, Daniel Zeller

**Affiliations:** ^1^Department of Neurology, University Hospital of Würzburg, Würzburg, Germany; ^2^Department of Neuroradiology, University Hospital of Würzburg, Würzburg, Germany

**Keywords:** multiple sclerosis, cerebellar tDCS, split-belt treadmill, locomotor adaptation, consolidation

## Abstract

**Background:** Multiple sclerosis (MS) may cause variable functional impairment. The discrepancy between functional impairment and brain imaging findings in patients with MS (PwMS) might be attributed to differential adaptive and consolidation capacities. Modulating those abilities could contribute to a favorable clinical course of the disease.

**Objectives:** We examined the effect of cerebellar transcranial direct current stimulation (c-tDCS) on locomotor adaptation and consolidation in PwMS using a split-belt treadmill (SBT) paradigm.

**Methods:** 40 PwMS and 30 matched healthy controls performed a locomotor adaptation task on a SBT. First, we assessed locomotor adaptation in PwMS. In a second investigation, this training was followed by cerebellar anodal tDCS applied immediately after the task ipsilateral to the fast leg (T0). The SBT paradigm was repeated 24 h (T1) and 78 h (T2) post-stimulation to evaluate consolidation.

**Results:** The gait dynamics and adaptation on the SBT were comparable between PwMS and controls. We found no effects of offline cerebellar anodal tDCS on locomotor adaptation and consolidation. Participants who received the active stimulation showed the same retention index than sham-stimulated subjects at T1 (*p* = 0.33) and T2 (*p* = 0.46).

**Conclusion:** Locomotor adaptation is preserved in people with mild-to-moderate MS. However, cerebellar anodal tDCS applied immediately post-training does not further enhance this ability. Future studies should define the neurobiological substrates of maintained plasticity in PwMS and how these substrates can be manipulated to improve compensation. Systematic assessments of methodological variables for cerebellar tDCS are urgently needed to increase the consistency and replicability of the results across experiments in various settings.

## Introduction

Multiple sclerosis (MS) is an inflammatory disease that affects the CNS by demyelination and neurodegeneration (Dobson and Giovannoni, 2019). There is a growing number of disease-modifying treatments available to modulate various aspects of the immunopathogenesis of MS (De Angelis et al., 2018). Although the clinical course of MS may be determined by the balance of disease activity on the one side and ability to show active resiliency on the other, the side of adaptation and compensation has received less attention so far. However, in view of the utmost importance of independent mobility for people with MS (PwMS) (Heesen et al., 2018), it appears vitally important to target those mechanisms making a stand against MS-related functional impairment.

A small body of literature suggests that PwMS are able to acquire motor skills despite impaired overall motor performance (Zeller et al., 2010; Bonzano et al., 2011; Tomassini et al., 2011). However, this capacity decreases with higher regional injury (Zeller et al., 2011). In the motor system, the acquisition of new skills requires a multistage process involving motor adaptation during which the movement is modified and calibrated from trial to trial based on error feedbacks. Following adaptation, motor memory is transformed from an initially fragile to a more robust state and therewith gains resistance to interference. This time-dependent process, called consolidation, determines the ability to recall and build upon adapted motor patterns across days and in new environmental conditions (Robertson et al., 2004; Krakauer and Shadmehr, 2006). Adaptation and consolidation would play a critical role in defining the degree of rehabilitation achieved by an individual with MS. Thus, the modulation of these abilities constitute an attractive strategy for successful rehabilitation.

With this background in mind, our analysis of recent studies showed that motor adaptation and consolidation can be modulated by non-invasive brain stimulation. For instance, tDCS is able to facilitate performance gains during motor training when applied over the primary motor cortex (Hummel et al., 2010; Hamoudi et al., 2018). Above this short-term effect, tDCS may facilitate consolidation when applied online during training (Reis et al., 2009, 2015) or offline immediately after motor training (Tecchio et al., 2010; Rumpf et al., 2017). Thus, anodal transcranial direct current stimulation (tDCS) applied on primary motor cortex soon after training (offline) improves consolidation of procedural learning in healthy subjects of young and older age groups (Tecchio et al., 2010; Zimerman et al., 2012; Rumpf et al., 2017). Moreover, anodal tDCS applied to the cerebellum increases the rate of adaptation to locomotor learning on the split-belt treadmill (SBT) (Jayaram et al., 2012). Notably, the cerebellum is thought to play a vital role in the dynamic regulation of balance, adaptation of posture and locomotion (Morton and Bastian, 2004).

We therefore aimed to examine locomotor adaptation and consolidation in PwMs using a split-belt paradigm. In order to modulate the consolidation processes, we applied anodal or sham tDCS to the cerebellum of the participants, offline immediately after the training. With this design, we would be able to disentangle the consequences of the split-belt training alone from the tDCS specific after-effects following the offline stimulation. Considerations that stimulation effects may be interfered by activity undertaken concurrently (Horvath et al., 2015) comforted the choice of an offline stimulation.

Specifically, we hypothesized that **(1)** the consolidation of locomotor adaptation is an important independent factor accounting for the preservation and recovery of motor function in PwMS, **(2)** locomotor consolidation is enhanced by cerebellar anodal tDCS compared to a sham intervention, particularly in patients with impaired consolidation.

## Materials and Methods

### Participants

This study was a single-center, double-blinded, parallel, randomized, sham-controlled design. The study conformed to the principles of the Declaration of Helsinki and was approved by the local Ethics Committee of the Medical Faculty at the University of Würzburg. All participants gave their written informed consent. They were naive to the purpose of the study.

The study consisted of two independent experiments with each subject taking part to only one experiment (experiment 1 without tDCS or experiment 2 with tDCS).

**FIGURE 1:**
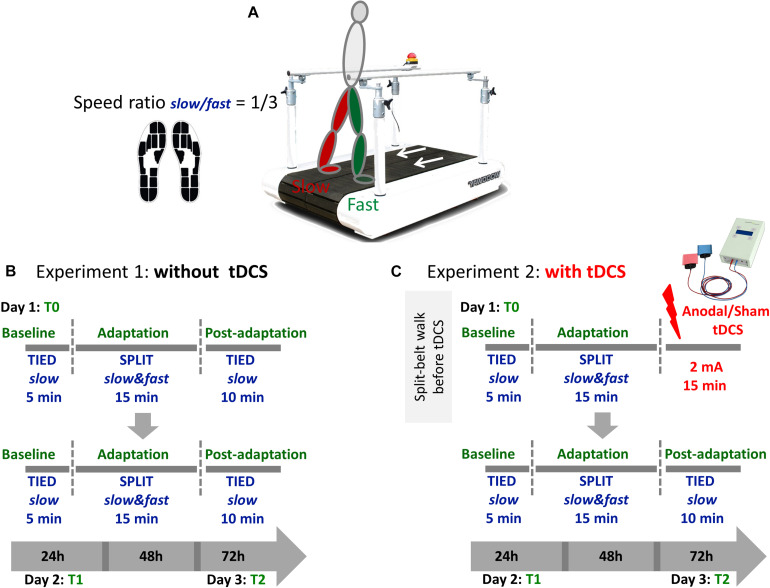
**(A)** Participants walked on a split-belt treadmill with a speed ratio 1/3 between the slow and the fast leg during the adaptation phase. Namely, the faster belt moved at the maximal speed obtained during the 25FWT and the slower belt always moved at the 1/3 of that speed. Gait parameters where recorded using sensor insoles. **(B)** Each session consisted of three phases (baseline, adaptation, and postadaptation). The clinically less affected leg (people with MS) or the dominant leg (controls) walked on the fast belt. During the baseline period, subjects walked with both belts tied at a slow speed for 5 min. During the adaptation period, they walked for 15 min in a split-belt condition, with one leg moving fast and the other slow. During the postadaptation period, subjects walked for 10 min with both belts tied at the slow speed. This training was repeated 24 h (T1) and 72 h (T2) after the first session. **(C)** In experiment 2, participants received anodal or sham tDCS over the cerebellum ipsilateral to the less affected/dominant leg immediately after the adaptation phase at T0 only. On the following days, the experiment was performed without tDCS.

The details about the experimental design are provided in section “Experimental Design” and Figure 1. To summarize, forty patients (23 female; aged 24–60 years) with definite MS following the McDonald criteria (Thompson et al., 2018), Expanded Disability Status Scale (EDSS) score between 1 and 6, were recruited from the outpatient clinic of the Neurology department at the University Hospital of Würzburg. Stable condition within the last 3 months and ability to perform the locomotor task were prerequisite to participate in the study. In addition, 30 age and gender-matched healthy controls (HC, 20 female, aged 26–60 years) took part in the study. None of the participants was taking centrally acting drugs.

### Clinical Assessment

All PwMS underwent a clinical assessment including (1) EDSS score (Kurtzke, 1983), (2) scale for the assessment and rating of ataxia (SARA) (Schmitz-Hubsch et al., 2006), (3) Würzburger Fatigue Inventory for MS (WEIMuS) (Flachenecker et al., 2006), (4) Beck Depression Inventory (BDI) (Beck et al., 1961), and Edinburgh Handedness Inventory (Oldfield, 1971). Lower limb function was assessed in HC and PwMS using timed walk tests (25 feet and 50 m) (Supplementary Table). Participants were pseudorandomly assigned to two experiments with or without tDCS. Regarding tDCS, PwMS and HC were pseudonymized and randomly assigned to anodal or sham cerebellar tDCS groups (Figure 1).

### CNS Injury

#### MRI Imaging

A total of 34 PwMS and 14 HC underwent a structural MRI scan (Siemens MAGNETOM Trio, 3.0 T scanner, Erlangen, Germany).

The sequence details were as follow:

• 2D TSE sequence: FoV (Field of View) = 240 mm, TR = 5000 ms, TE = 101 ms, in plane resolution 0.5 × 0.5 mm, 3 mm slice thickness.
• 2D FLAIR: FoV = 240 mm, TR = 8000 ms, TE = 135 ms, TI = 2320 ms, in plane resolution 0.9 × 0.9 mm, 3 mm slice thickness.
• 3D CISS (Constructive Interference in Steady State): FoV = 180 mm, TR = 7.72 ms, TE = 3.34 ms, in plane resolution 0.4 × 0.4 mm, 3 mm slice thickness.
• MPRAGE (TFL): FoV = 256 mm, TR = 2530 ms, TE = 3.37 ms, TI = 1200 ms, FA (Flip angle) = 7°, 1 × 1 × 1 mm^3^ isotropic voxel size.
• Single voxel spectroscopy (SVS SE): TR = 2000 ms, TE = 135 ms, 80 averages, FA = 90°, 20 × 20 × 20 mm^3^ voxel size, water suppression band width = 50 Hz.

#### MRI Analysis

The lesions on T2-weighted FLAIR sequences were outlined by a semi-automated edge finding software tool and, where necessary, corrected manually by the investigator blind to the behavioral assessment of the study. In addition, supra- and infratentorial lesion load were assessed separately. To complete the structural analysis, high resolution scans (3D CISS) were acquired to evaluate C2 spinal cord cross-sectional area. Finally, MR-spectroscopy was performed to determine the absolute *N*-acetyl-aspartate (NAA) integral (with TARQUIN^1^) in a single-voxel MRS measurement (centered on each cerebellar hemisphere).

#### Motor Evoked Potentials

The functional integrity of the corticospinal tract in PwMS was tested by evaluating the central motor latency of the motor evoked potential of the tibialis anterior (TA) muscle bilaterally using transcranial magnetic stimulation (TMS) (Claus, 1990). The procedure corresponded to the clinical routine for the diagnostic of MS. Briefly, subjects were seated in a comfortable chair with arms resting on a cushion and the legs stretched on another chair in front of them. Surface electromyography (EMG; Evidence 3102 evo, Neurosoft, Russia), was recorded from the TA in a belly-tendon montage (reference electrode over the tibia at ankle level). EMG signal was amplified, band-pass filtered (10 Hz to 2 kHz) and sampled at 10 kHz. TMS of the motor cortex and the ventral root at the spinous processes between L4 and L5 was undertaken using a round magnetic coil at 100% of the stimulator output (Neuro-MS TMS device, Neurosoft, Russia). Cortical stimulation was applied during voluntary contraction of the TA, while root stimulation was applied with the muscle at rest. Each hemisphere was stimulated in turn, by turning the coil over in order to reverse the direction of current flow. The central motor conduction time was calculated by subtracting the latency to onset of the EMG response to each muscle for ventral root stimulation from that for cortical stimulation. To be able to perform group comparisons, the individual latency was corrected to body height (Zeller et al., 2014).

### Experimental Design

#### Split-Belt Treadmill Paradigm

Subjects participated in a locomotor adaptation task (LAT) on a SBT (Woodway, Waukesha, WI, United States) (Reisman et al., 2005; Jayaram et al., 2011, 2012) with belts moving together (tied-*slow*) or at different speeds (split-*slow/fast*). Figure 1 shows the experimental paradigm. The less affected or the dominant leg was made to move faster during the adaptation phase. For all testing, subjects wore comfortable walking shoes and a safety harness (Figure 1A).

In order to assess motor learning and consolidation, participants were retested 24 h (T1) and 72 h (T2) after the beginning of the training session.

#### Data Collection

Gait parameters were recorded using special insoles consisting of 13 pressure sensors and a 3D acceleration sensor (Moticon Insoles, Munich, Germany) (Figure 1A). These insoles have been validated in the clinical setting (Braun et al., 2015, 2016). The gaitline length (mm), that is path of the center of pressure (CoP) under the foot during one ground contact phase, was collected for each step to complete motion analysis.

#### Cerebellar tDCS – Only for Experiment 2

Immediately at completion of the training on the first day (T0), offline tDCS was delivered through two 25 cm^2^ surface (5 × 5) saline-soaked sponge electrodes to the subjects in experiment 2. Both the participants and the experimenter were blinded to the type of stimulation (sham or anodal) using the “study mode” implemented in the tDCS device (DC-Stimulator-Plus, Neuroconn, Germany). Subjects were randomized to receive anodal or sham tDCS over the cerebellar hemisphere ipsilateral to *fast* leg with the anodal electrode applied over the cerebellum 3 cm lateral to the inion and the cathodal electrode positioned on the ipsilateral buccinator muscle (Jayaram et al., 2012). At the onset of stimulation, current was increased in a ramp-like fashion over a period of 10 s. In the anodal group, a 2 mA current (current density 0.08 A/cm^2^) was applied for 15 min. Sham stimulation started identically with a short linear fade-in phase, followed by 2 mA of direct current for 30 s and a short linear fade-out phase. During the main time of stimulation, only small current pulses occurred every 550 ms (110 μA over 15 ms). This procedure has been shown to ensure the blinding of the subjects (Gandiga et al., 2006).

### Data Analysis

Gait data were cropped into baseline, adaptation and postadaptation. Only the last 100 strides performed in the baseline phase were analyzed in order to exclude the acclimation period from evaluation. A total of 600 strides in the adaptation and 150 strides in the postadaptation phase were analyzed. The accuracy of stride detection was individually verified by visual inspection.

The gaitline lengths (mm) for the slow and the fast foot were extracted. To evaluate adaptation during the locomotor task on the SBT and its subsequent consolidation, the symmetry of the gaitline length during the late baseline, the adaptation and the postadaptation phase, was calculated as follows:

$$Gaitlinelengthsymmetry\;=\frac{\mathrm{Gaitline}\,\mathrm{length}\,f\,a\,s\,t-\mathrm{Gaitline}\,\mathrm{length}\,s\,l\,o\,w}{\mathrm{Gaitline}\,\mathrm{length}\,f\,a\,s\,t+\mathrm{Gaitline}\,\mathrm{length}\,s\,l\,o\,w}$$

The results were grand-averaged across participants of the same group before proceeding with further analysis.

We defined the retention index (RI) as a read-out of successful consolidation. This rate was calculated as the ratio of the average gaitline length symmetry in early adaptation (EA) on the time-point evaluated (T1 or T2) to the average gaitline length symmetry in the late adaptation (LA) of the previous time-point (here T0 or T1):

$$\mathrm{RI}=\frac{\mathrm{EA}\,T\,\left(x\right)}{\mathrm{LA}\,T\,\left(x-1\right)}$$

To assess the relationship between baseline characteristics of the participants in experiment 1 (EDSS score, 25FWT, CNS injury) and the retention rate, data were split into (i) PwMS with EDSS≤2 and PwMS with EDSS > 2 (Comber et al., 2017) or (ii) 25FWT≤5.2 s and 25FWT > 5.2 s (Phan-Ba et al., 2011).

### Statistics

#### Sample Size Calculation

At the beginning of this study there were, to our knowledge, neither studies comparing locomotor adaptation between PwMS and controls, nor data assessing the effect of offline cerebellar tDCS on locomotor consolidation in PwMS. In order to estimate the number of participants necessary to achieve a power of 0.8, we applied the *a priori* power analysis for *F*-test family using the ANOVA for repeated measures within-between interactions in order to match our mixed model ANOVA design (with *group* [PwMS, controls] as between factor and *day* [T0, T1, T2] as within-factor). For this estimation, we used the software GPower 3.1 (Heinrich Heine Universität Düsseldorf). We obtained an estimation of 54 participants for the study. This would mean a minimum of nine participants for each of the six groups. This number was increased to 15 for the patients receiving tDCS (PwMSsham, PwMSreal) in order to account for the expectable heterogeneity of the response in that population.

#### Main Outcome Measure

The average gaitline length symmetry of the last six strides in the baseline, the first and last six strides of the adaptation period (*early*, EA and *late adaptation*, LA, respectively), and the first six strides of the postadaptation period (*early postadaptation*, EPA) was considered in the current analysis. We also defined *mid-adaptation*, MA, as the average of the 50 strides following EA in order to evaluate the mid-course of gaitline length symmetry during the split period.

#### Statistical Analysis

The Shapiro–Wilk test was used to test for normality. Descriptive and clinical data were given as mean ± SD or median [range] for ordinal data. Unpaired Student’s *t*-test was used to compare clinical, demographic and CNS injury data in experiment 1 (HC, PwMS). The one-way analysis of variance (ANOVA) was used for multiple group comparisons in experiment 2 (HCsham, HCreal, PwMSsham, PwMSreal). The data collected for EDSS score, age, disease duration and CNS injury were not normally distributed in experiment 2. We used the non-parametric Mann–Whitney *U* rank test to compare EDSS score and disease duration in between PwMSsham and PwMSreal. The Kruskal–Wallis test was applied to compare age and CNS injury between HCsham, HCreal, PwMSsham, and PwMSreal.

Mixed model ANOVA with *post hoc* comparisons were performed to assess the change in gait symmetry during EA, MA, LA, and EPA at T0, T1, and T2. Sphericity was tested using the Mauchly test and a Greenhouse–Geisser correction was applied when appropriate. Two-tailed Pearson’s and Spearman’s correlations were used to assess associations between baseline clinical characteristics and retention rate. Bonferroni correction was applied for multiple comparisons. The significance level was set at *p* < 0.05.

## Results

### Demographics and Clinical Characteristics

Details about the clinical and demographic features of PwMS and HC are given in the Supplementary Table.

*Experiment 1 (SBT training without tDCS)* included 10 PwMS (7 females) and 10 HC (7 females). There was no difference between HC and PwMS with regards to age [*t*(18) = −0.09, *p* = 0.93]. HC performed significantly better than PwMS on the T25FWT [*t*(18) = −2.08, *p* = 0.05]. EDSS scores correlated with performance on the T25FWT (*r* = 0.83, *p* = 0.003), but not with disease duration (*r* = −0.006, *p* = 0.99) as expressed by the Spearman correlation.

*Experiment 2 (SBT training with cerebellar anodal tDCS)* included two main groups of 30 PwMS (16 females) and 20 HC (13 females). Within their groups, participants were randomly assigned to one of two possible cerebellar tDCS modes: HC-sham, HC-real, PwMS-sham, PwMS-real. A Kruskal–Wallis *H* test showed that there was no statistical difference in age between groups, *X*^2^(3) = 1.19, *p* = 0.76. We found no difference in the T25FWT, *X*^2^(3) = 6.4, *p* = 0.094 between HC and PwMS. PwMS were only mildly affected. EDSS score (*U* = 90, *p* = 0.37) and disease duration (*U* = 94, *p* = 0.46) of PwMS sham and PwMS real were comparable.

In PwMS sub-groups, EDSS score correlated significantly with T25FWT (*sham*: *r* = 0.58, *p* = 0.022, *real*: *r* = 0.67, *p* = 0.006) and disease duration (*sham*: *r* = 0.5, *p* = 0.05, *real*: *r* = 0.65, *p* = 0.009).

### CNS Injury

Evaluation of CNS injury for PwMS and controls is summarized in Table 1.

**TABLE 1:** CNS injury was evaluated using the central motor latency (CML) of lower limb and MRI measures.

**Experiment 1**				
**Groups**	**HC**	**PwMS**		
CML_left	–	−0.71 ± 2.17 (*n* = 10)		
CML_right	–	−0.14 ± 2.79 (*n* = 10)		
ΔCMCT	–	−0.52 ± 1.47 (*n* = 10)		
Ventr. lesion (mm^3^)	128.93 ± 66.88 (*n* = 10)	6903.28 ± 2117.29** (*n* = 10)		
Non-ventr. lesion (mm^3^)	172.96 ± 72.53 (*n* = 5)	3170.78 ± 920.43* (*n* = 9)		
Total lesion (mm^3^)	430.83 ± 121.89 (*n* = 5)	10841.02 ± 2703.04* (*n* = 9)		
C2 inner diameter	1922.85 ± 100.91 (*n* = 5)	1772.18 ± 83.96 (*n* = 9)		
C2 outer diameter	5752.08 ± 367.42 (*n* = 5)	5248.85 ± 112.25* (*n* = 9)		
NAA/Cr. ratio	1.242 ± 0.04 (*n* = 5)	1.19 ± 0.04 (*n* = 9)		
**Experiment 2**				
**Groups**	**HC sham**	**HC real**	**PwMS sham**	**PwMS real**
CML_left	–	–	−0.24 ± 2.30 (*n* = 15)	−0.18 ± 2.25 (*n* = 15)
CML_right	–	–	−1.73 ± 2.95 (*n* = 15)	−0.55 ± 1.35 (*n* = 15)
ΔCMCT	–	–	1.49 ± 1.05 (*n* = 15)	−1.39 ± 1.45 (*n* = 15)
Ventr. lesion (mm^3^)	113.38 ± 58.75 (*n* = 8)	182.98 ± 1227.88 (*n* = 10)	7017.53 ± 2308.56**** (*n* = 15)	5223.76 ± 2589.20**** (*n* = 14)
Non-ventr. lesion (mm^3^)	0.00 (*n* = 5)	111.40 ± 50.76 (*n* = 4)	1945.66 ± 485.78*** (*n* = 14)	1391.19 ± 347.50*** (*n* = 11)
Total lesion (mm^3^)	181.4 ± 81.11 (*n* = 5)	568.86 ± 265.64 (*n* = 4)	9464.44 ± 2761.44*** (*n* = 14)	8039.62 ± 3254.56*** (*n* = 11)
C2 inner diameter	1801.58 ± 64.02 (*n* = 5)	1973.12 ± 38.9 (*n* = 4)	1892.41 ± 53.47 (*n* = 14)	1778.91 ± 58.48 (*n* = 11)
C2 outer diameter	4760.87 ± 441.16 (*n* = 5)	5038.14 ± 208.02 (*n* = 4)	4745.68 ± 250.94 (*n* = 14)	4374.65 ± 306.10 (*n* = 11)
NAA/Cr. ratio	1.23 ± 0.13 (*n* = 5)	1.20 ± 0.07 (*n* = 4)	1.10 ± 0.09 (*n* = 14)	1.20 ± 0.03 (*n* = 11)

Negative value in CML indicates increased latency. The volumes of ventricular, non-ventricular and total cerebral lesions are expressed as mean ± SD. The diameter of the spinal cord at the C2 level as well as the ratio of *N*-acetylaspartate/creatine brain metabolites (NAA/Cr) are presented. Levels of significance for differences between controls and PwMS are *p < 0.05, **p < 0.01, ***p < 0.001, and ****p < 0.0001.

*Experiment 1 (SBT training without tDCS)* indicated significantly higher ventricular [*t*(18) = −3.2, *p* = 0.005], periventricular [*t*(12) = −2.4, *p* = 0.035] and total lesion volume [*t*(12) = −2.82, *p* = 0.016] in the brain of PwMS (*N* = 9) compared to HC (*N* = 5). There were no differences in the C2 inner diameter [*t*(12) = 1.11, *p* = 0.29] and C2 outer diameter [*t*(12) = 1.65, *p* = 0.13]. We found similar NAA/Cr ratios in PwMS and HC [*t*(12) = 0.86, *p* = 0.41]. Only two patients presented a pathological central motor latency for the lower limb.

*Experiment 2 (SBT training with cerebellar anodal tDCS)*: We found significantly higher ventricular [*X*^2^(3) = 19.94, *p* < 0.0001], periventricular [*X*^2^(3) = 17.36, *p* = 0.001] and total lesion volume [*X*^2^(3) = 15.83, *p* = 0.001] in PwMS compared to HC. C2 inner and outer diameter did not differ between groups [*X*^2^(3) = 1.62, *p* = 0.65 and *X*^2^(3) = 4.37, *p* = 0.22, respectively]. Also the NAA/Cr ratios were comparable between groups [*X*^2^(3) = 0.73, *p* = 0.87].

Four patients in PwMS-sham and six patients in PwMS-real presented pathological central motor latency for the lower limb. There were no significant differences in central motor latency between the two groups on the left leg (*U* = 108.5, *p* = 0.87) and the right leg (*U* = 105, *p* = 0.78) denoting a similar level of pyramidal demyelination for the individuals with prolonged central motor conduction time.

### Evaluation of Locomotor Consolidation in PwMS

**FIGURE 2:**
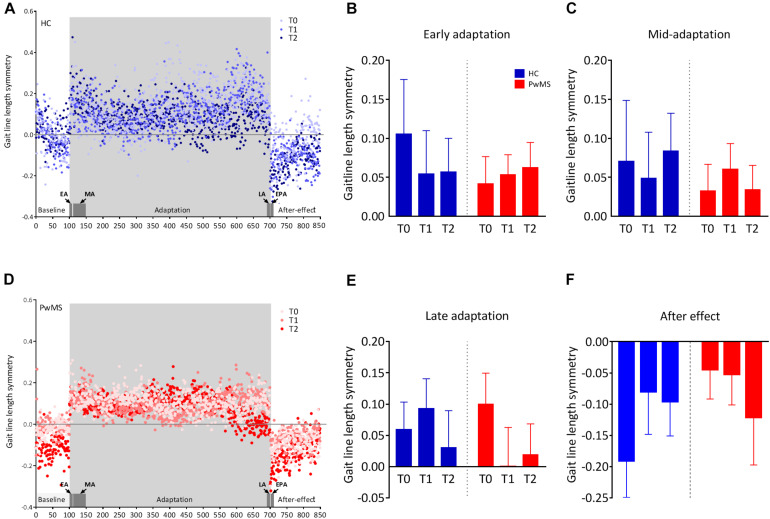
Changes in the symmetry of gaitline length across training phases in experiment 1 (training without tDCS): **(A,D)** The changes in the mean gaitline symmetry for each training phase are illustrated with respect to the stride number for healthy controls (HC) and people with multiple sclerosis (PwMS). The first and last six strides of the adaptation period define the *early*, EA and the *late adaptation*, LA, respectively. The first six strides of the postadaptation period are called *early postadaptation*, EPA and the *mid-adaptation*, MA is the average of the 50 strides following EA. Dots represent the means of the calculated gaitline symmetry at each stride for the entire group (HC or PwMS). Training on the split-belt treadmill was performed at T0, T1 (24 h after the first trial) and T2 (72 h after the first trial). The blue color represents data from HC and red dots are data obtained from PwMS. **(B)** Average gaitline length symmetry in the early adaptation obtained from the first six strides in the adaptation phase. **(C)** Average gaitline length symmetry during mid-adaptation obtained from the first 50 strides following early adaptation in the adaptation phase. **(E)** Average gaitline length symmetry during late adaptation obtained from the last six strides in the adaptation phase. **(F)** Average gaitline length symmetry during early postadaptation obtained from the first six strides in the postadaptation phase of the training. Error bars represent the standard error of means (sem, *N* = 10 per group).

All participants were able to complete the walking task on the SBT. We observed a walking behavior that has been previously described (Reisman et al., 2005). In short, the initial symmetry of gaitline length in the baseline phase shifted to a large asymmetry (positive values) when the speed between the left and the right leg was split. This change in the early adaptation rapidly returned to baseline symmetry (close to zero) after ∼150 steps, indicating successful adaptation. After the adaptation phase, the belts were tied at the baseline speed (postadaptation phase), which resulted in an aftereffect consisting of an asymmetric gait in the opposite direction to the early adaptation (Figures 2A,D).

At baseline of experiment 1, mixed-model ANOVA with the between-subject factor *group* (HC vs. PwMS) and the within factor *day (T0, T1, T2)* showed no significant *group × day* interaction indicating similar minimal error in baseline blocks as function of day between groups. There was no significant main effect of *group* at baseline underlining the absence of initial differences between groups (*p* = 0.59). When the speed between the left and right limb was different, both HC and PwMS showed an initial gaitline deviation that was highly variable throughout the adaptation period. There was no significant *group × day* interaction and no main effect of *day or group* on the gaitline length symmetry at EA, MA, and LA (Figures 2B,C,E and Table 2). When the speed was set back to baseline in the postadaptation period, both HC and PwMS showed an initial reverse asymmetry (after effect). However, we found no significant *group × day* interaction and no main effect of *day* or *group* on the calculated gaitline length symmetry at EPA (Figure 2F and Table 2).

**TABLE 2:** Two-way mixed-model ANOVA of gaitline length symmetry across training phases (BSL, late baseline; EA, early adaptation; MA, mid-adaptation; LA, late adaptation; EPA, early postadaptation), with the between-subject factor *group* (HC vs. PwMS) and the within factor *day* (T0, T1, T2).

**Source**	**Correction**	**Df**	***F***	***p***	**$\eta_{\text{p}}^{2}$**
**Within-group effects BSL**	GG				
Day (T0, T1, T2)		1.451	0.046	0.909	0.003
Day × group		1.451	1.548	0.231	0.079
Error day		26.123			
**Between-group effects BSL**					
Group		1	0.297	0.593	0.016
Error group		18			
**Within-group effects EA**	GG				
Day (T0, T1, T2)		1.316	0.238	0.696	0.015
Day × group		1.316	0.677	0.459	0.041
Error day		32			
**Between-group effects EA**					
Group		1	0.139	0.714	0.009
Error group		16			
**Within-group effects MA**					
Day (T0, T1, T2)		2	0.019	0.981	0.001
Day × group		2	0.392	0.679	0.001
Error day		30			
**Between-group effects MA**					
Group		1	0.299	0.593	
Error group		15			
**Within-group effects LA**					
Day (T0, T1, T2)		2	0.501	0.613	0.044
Day × group		2	3.099	0.065	0.220
Error day		22			
**Between-group effects LA**					
Group		1	0.604	0.454	
Error group		11			
**Within-group effects EPA**					
Day (T0, T1, T2)		2	0.697	0.506	0.042
Day × group		2	0.303	0.733	0.019
Error day		32			
**Between-group effects EPA**					
Group		1	0.009	0.926	
Error group		16			

**p* < 0.05 is significant; GG, Greenhouse–Geisser correction, *N* = 10 per group.*

In summary, during SBT locomotion, HC and PwMS were equally able to rapidly change their walking pattern to accommodate different belt speeds at T0. This ability was maintained but not improved through the task repetition at T1, T2.

**FIGURE 3:**
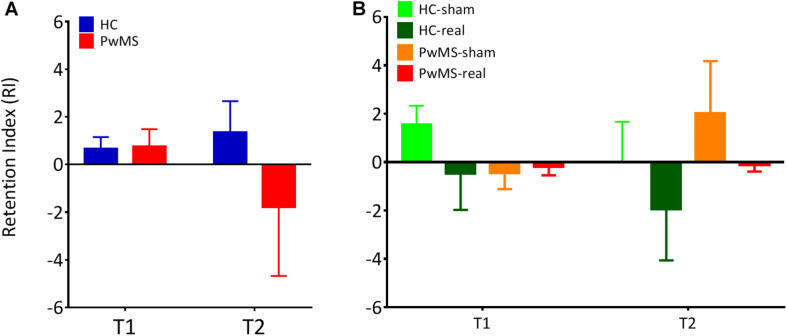
The retention index was calculated for T1 and T2 following the first training session. This value is defined as the ratio of the average gaitline length symmetry in the early adaptation (EA) at the respective day to the average gaitline length symmetry in the late adaptation phase (LA) of the previous day. **(A)** Retention index for experiment 1 without tDCS (*N* = 10 per group). **(B)** Retention index for experiment 2 with tDCS (*N*_HC–sham_ = 9, *N*_HC–real_ = 11, *N*_PwMS–sham_ = 15, *N*_PwMS–real_ = 15). Error bars represent the standard error of means.

In order to confirm this result, we calculated the RI of the learned task between sessions. We found no significant difference in RI between HC and PwMS at both T1 (*p* = 0.76) and T2 (*p* = 0.35) indicating similar consolidation level in both groups (Figure 3A and Table 3, upper part). However, we observed a high RI variability in the group analysis of PwMS.

**TABLE 3:** Comparison of the in retention index (RI) between the groups (Student *t*-test for independent samples in experiment 1, one-way ANOVA in experiment 2).

**Variable**	**Time**	**Group**	***N***	**Mean**	**SD**	***t***	***p***
RI- Experiment 1	T_1_	HC	10	1.12	1.88		
		PwMS	10	0.77	2.22	0.32	0.76
	T_2_	HC	10	1.51	4.32		
		PwMS	10	−1.98	9.67	0.99	0.35
**Variable**	**Time**	**Group**	***N***	**Mean**	**SD**	***F***	***p***
RI- Experiment 2	T_1_	HC sham	9	1.6	2.57		
		HC real	11	−0.43	4.5		
		PwMS sham	15	−0.46	2.55	1.18	0.33
		PwMS real	15	−0.21	1.31		
	T_2_	HC sham	9	0.01	1.88		
		HC real	11	−1.76	1.93		
		PwMS sham	15	2.02	2.15	0.88	0.46
		PwMS real	15	−0.13	0.26		

**p* < 0.05 is significant.*

This variability was independent from MS-specific clinical parameters as we found no correlation between RI at T2 and CNS injury in PwMS stratified by gait impairment with T25FWT ≤ 5.2 s (*r* = 0.14, *p* = 0.79) and T25FWT > 5.2 (*r* = 0.16, *p* = 0.84). Similarly, RI at T2 did not correlate with CNS injury in PwMS stratified by EDSS score ≤ 2 (*r* = 0.74, *p* = 0.33) and EDSS score > 2 (*r* = 0.03, *p* = 0.9).

### Effect of tDCS on Locomotor Consolidation in PwMS

In experiment 2, cerebellar tDCS was applied immediately after the adaptation period at T0. The stimulation was well tolerated without discomfort and no participant reported adverse effects following the session. Comparisons of gait symmetry during the testing periods (baseline, EA, MA, LA, EPA) are summarized in Table 4.

**TABLE 4:** Two-way mixed-model ANOVA of gaitline length symmetry across training phases (BSL, late baseline; EA, early adaptation; MA, mid-adaptation; LA, late adaptation; EPA, early postadaptation) Mixed model ANOVA with the between-subject factor *group* (HC-sham, HC-real, PwMS-sham, PwMS-real) and the within factor *day* (T0, T1, T2).

**Source**	**Correction**	**Df**	***F***	***p***	**$\eta_{\text{p}}^{2}$**
**Within-group effects BSL**	GG				
Day (T0, T1, T2)		1.704	1.213	0.298	0.026
Day × group		5.111	0.139	0.984	0.009
Error day		92			
**Between-group effects BSL**					
Group		3	0.78	0.459	0.054
Error group		46			
**Within-group effects EA**					
Day (T0, T1, T2)		2	0.197	0.821	0.005
Day × group		6	0.120	0.994	0.010
Error day		37			
**Between-group effects EA**					
Group		3	0.222	0.881	
Error group		37			
**Within-group effects MA**	GG				
Day (T0, T1, T2)		1.687	0.269	0.765	0.006
Day × group		5.061	0.460	0.836	0.006
Error day		43			
**Between-group effects MA**					
Group		3	0.677	0.571	
Error group		43			
**Within-group effects LA**					
Day (T0, T1, T2)		2	0.258	0.774	0.014
Day × group		6	0.934	0.483	0.135
Error day		36			
**Between-group effects LA**					
Group		3	0.523	0.672	
Error group		11			
**Within-group effects EPA**					
Day (T1, T2)		1	0.134	0.716	0.003
Day × group		3	0.597	0.620	0.037
Error day		46			
**Between-group effects EPA**					
Group		3	0.782	0.070	
Error group		46			

**p* < 0.05 is significant. GG, Greenhouse–Geisser correction. *N*_HC–sham_ = 9, *N*_HC–real_ = 11, *N*_PwMS–sham_ = 15, *N*_PwMS–real_ = 15.*

**FIGURE 4:**
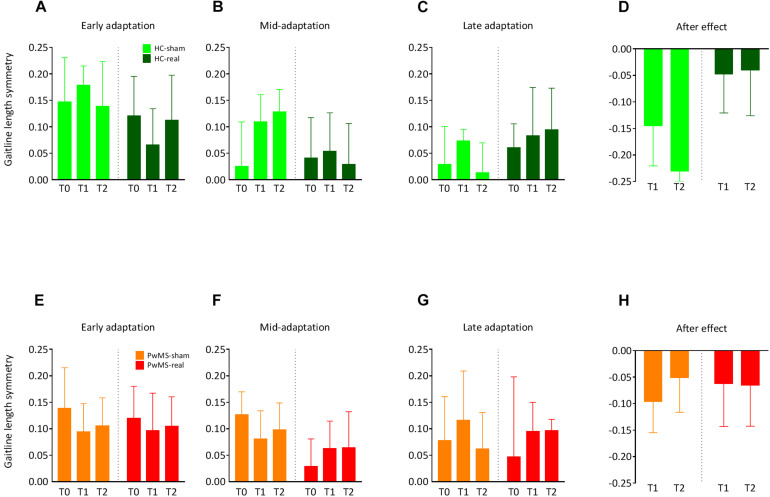
Changes in the symmetry of gaitline length across training phases in experiment 2 (with tDCS): Training on the split-belt treadmill was performed at T0, T1 (24 h after the first trial), and T2 (72 h after the first trial). Cerebellar anodal (real) or sham tDCS was applied ipsilateral to the *fast* leg immediately after the adaptation phase at T0. The blue color represents data from HC, data obtained from PwMS are red-colored. **(A,E)** Average gaitline length symmetry in the early adaptation obtained from the first six strides in the adaptation phase for HC and PwMS. **(B,F)** Average gaitline length symmetry during mid-adaptation obtained from the first 50 strides following early adaptation in the adaptation phase. **(C,G)** Average gaitline length symmetry during late adaptation obtained from the last six strides in the adaptation phase. **(D,H)** Average gaitline length symmetry during early postadaptation obtained from the first six strides in the postadaptation phase of the training, only at T1 and T2. Error bars represent the standard error of means (*N*_HC–sham_ = 9, *N*_HC–real_ = 11, *N*_PwMS–sham_ = 15, *N*_PwMS–real_ = 15).

There was no relevant *group × day* interaction indicating similar gaitline length symmetry as function of day between both groups (*p* = 0.3) at baseline. There was no main effect of *day* (*p* = 0.3) or *group* (*p* = 0.46) underlining the absence of initial differences between groups independently from the day of experiment. At EA, ANOVA showed no *group × day* interaction (*p* = 0.99), no main effect of *day* (*p* = 0.82), and no main effect of *group* (*p* = 0.88) (Figures 4A,E and Table 4). Similarly, there were no relevant *group × day* interactions (*p* = 0.48) and no main effects of *day* or *group* (*p* = 0.67) at LA (Figures 4C,G and Table 4).

The LA was immediately followed by cerebellar tDCS at T0. Participants performed the postadaptation phase at T1 and T2. We compared the gaitline length symmetry at EPA. HC and PwMS showed similar magnitude of aftereffects at T1 and T2 independently from the stimulation mode (sham vs. real). There was no *group × day* interaction (*p* = 0.62). We found no main effect of *day* (*p* = 0.72). However, ANOVA indicated a trend for the main factor group (*p* = 0.07) driven by a greater after effect in the HC-sham group compared to HC-real, PwMS-sham and PwMS-real groups (Figures 4D,H and Table 4). The absence of tDCS effect on EA when only the first six strides were averaged led us to perform an analysis on the following 50 strides in the split phase at T0, T1 and T2 in order to evaluate the initial difference in adaptation between HC and PwMS at the beginning of each testing day (mid-adaptation, MA). Similarly to EA, variance analysis revealed no significant *group × day* interaction (*p* = 0.84) no significant main effects of *day* (*p* = 0.77) and *group* (*p* = 0.57) at MA (Figures 4B,F and Table 4).

In summary, during SBT locomotion, HC and PwMS were equally able to rapidly change their walking pattern to accommodate different belt speeds at T0. Cerebellar anodal tDCS did not modulate gait behavior at T1 and T2 in HC and PwMS compared to sham stimulation.

In order to confirm this result, we calculated the RI of the learned task from T0 to T1 and T1 to T2. One-way ANOVA showed no significant difference in RI between HC-sham, HC-real, PwMS-sham and PwMS-real at both T1 and T2, indicating similar consolidation levels in both groups (Figure 3B and Table 3, lower part).

## Discussion

We examined locomotor adaptation in PwMS using a split-belt paradigm (experiment 1) and investigated the effects of cerebellar tDCS, applied offline immediately after the training, on the consolidation of the learnt skill (experiment 2). In contrast to our hypotheses, PwMS showed similar behavior on the SBT compared to HC, independently from their lesion load and their motor function. Furthermore, there were no effects of cerebellar tDCS on the consolidation process when comparing sham to anodal stimulation both in HC and in PwMS.

If we postulate that neuronal plasticity represents an important substrate for the success of locomotor adaptation on the SBT, our results do not support the hypothesis that mechanisms of brain recovery that underlie this form of adaptation are substantially impaired in PwMS within the disease burden and disability range studied here.

### Locomotor Adaptive Learning and Consolidation in PwMS

To our knowledge, this study is the first to investigate the effect of a split-belt paradigm on motor adaptation and consolidation in PwMS. Our patients were mildly affected according to their EDSS score. During SBT locomotion, PwMS were able to rapidly change their walking pattern to accommodate the change in belt’s speed. This change indicated the ability of PwMS to successfully express adaptation and aftereffect by varying the asymmetry between their left and right foot gaitline length. Our observation agrees well with studies demonstrating learning capacities in PwMS using a variety of different tasks such as finger tapping (Mancini et al., 2009), visuomotor tracking (Tomassini et al., 2011), and simple force production (Zeller et al., 2010). However, those studies focused on upper-limb function while lower limbs have received little attention.

PwMS can scale their postural responses when exposed to predictable surface perturbations on a standing platform (Cameron et al., 2008). Short-term adaptations driven by an error augmentation strategy on the SBT have been shown to significantly reduce asymmetry in stroke patients after 12 training sessions (Reisman et al., 2013). Those aftereffects lasted up to 1 month (Reisman et al., 2009). We couldn’t reproduce this long-term consolidation effect in PwMS. As opposed to hemiparetic stroke patients where the paretic step is usually longer than the opposite step (Reisman et al., 2009), the population of PwMS investigated here did not present with initial gait asymmetry. Therefore, the differential effects of repeated SBT on PwMS relative to stroke patients might indicate differential biomechanical strategies of locomotor adaptation to split-belt walking between the two groups. The CNS injury and the level of disability as measured by the EDSS score or the 25FWT did not correlate with the retention index of the task. This result further confirmed that the disability levels did not predict the actual skill learning process in PwMS on the SBT.

Overall, an important finding of this study is that PwMS with mild-to-moderate impairment – despite MS-related CNS damage – are still able to display the plasticity required to make reactive and predictive adjustments in a changing environment.

### Anodal Cerebellar tDCS Effects on Locomotor Adaptation Task

Cerebellar integrity is critical for trial-and-error adaptation of motor behaviors to new contexts (Morton and Bastian, 2004). Cerebellar damage significantly disrupts feedforward motor adaptation during SBT locomotion (Morton and Bastian, 2006). Locomotor learning has also been associated with a decrease of cerebellar inhibition over the motor cortex (Jayaram et al., 2011) underlying that this type of adaptive learning is mediated, at least partly, by long-term depression in Purkinje Cells. The idea is also supported by animal studies showing the association of long-term depression in Purkinje cells with adaptive learning on one hand (Medina and Lisberger, 2008) and the direct implication of the cerebellum and not the cerebral cortex on locomotor adaptation on the other hand (Darmohray et al., 2019). In the same line, greater cortico-cerebellar connectivity has been correlated with learning performance in a finger-tapping task in PwMS (Bonzano et al., 2015). This opens the opportunity to specifically target the cerebellum to enhance motor learning. To modulate cortico-cerebellar connectivity, we applied cerebellar tDCS immediately after the training at T0. Gait adaptation was similar for controls and PwMS independently of the stimulation mode (sham/anodal). Until now, most of the studies looking at the effect of tDCS on consolidation, have applied the stimulation during the task. tDCS-induced facilitation of consolidation has also been previously achieved with offline stimulation over the primary motor cortex after completion of the training (Tecchio et al., 2010; Rumpf et al., 2017) or the cerebellum using anodal (Galea et al., 2009) and cathodal polarization (Pope and Miall, 2012).

However, there are many inconsistencies regarding the impact of cerebellar tDCS on adaptation in various tasks. Recent studies have reported cerebellar tDCS having no effect on adaptation in a dynamic balance task (Steiner et al., 2016), visuomotor adaptation (Jalali et al., 2017), or force field adaptation (Mamlins et al., 2019). Here, we found no effects of the offline stimulation on locomotor consolidation.

Jayaram et al. (2012) had shown a positive effect of concurrent application of cerebellar anodal tDCS with SBT walking. In this study, we analyzed offline effects of cerebellar tDCS and cannot directly compare both results. Indeed, tDCS effects are timing-dependent. For instance, anodal tDCS applied during the task can speed motor learning whereas its application before the task slows motor learning in an explicit sequence learning task (Stagg et al., 2011). Similarly, applying cerebellar anodal tDCS during motor task could increase skill acquisition, while improving consolidation when applied after a two-hand coordination task (Beck et al., 1961). We had expected that upregulating the activity of cerebellar cortical neurons by applying anodal tDCS after the locomotor task would increase subsequent motor consolidation. Unfortunately, this hypothesis was not confirmed by our results.

In a work investigating the effects of cerebellar tDCS on adaptation in a dynamic balance task, subjects who concurrently received verum stimulation tended to perform worse compared to sham (Steiner et al., 2016). Lack of effects or even negative effects of cerebellar tDCS are increasingly reported in the literature (Westwood et al., 2017; Mamlins et al., 2019). However, it is very difficult to determine a true effect size not only for cerebellar tDCS, but also for tDCS in general due to the clear publication bias toward positive effects in the literature.

### Limitations and Future Directions

In our study, the effect of cerebellar tDCS was evaluated in a sample size between 9 and 15 participants per group estimated after a power analysis. This size is in the range of previously published papers (Galea et al., 2011; Jayaram et al., 2011; Hardwick and Celnik, 2014). However, Minarik et al. (2016) showed that with a suggested tDCS effect size of 0.45, the likelihood of observing a significant result with 14 participants (per group) was only 20%. Jalali et al. (2017) have pooled data across several experiments to increase the tDCS effect size. Yet, they were unable to reproduce their own previously published results.

We evaluated the effect of a single tDCS stimulation on locomotor consolidation. The absence of effects observed in the present study is therefore limited to that context. On one side, past works using single anodal cerebellar tDCS have found an effect of stimulation on motor adaptation (Jayaram et al., 2012) and other studies did not show such an effect (Steiner et al., 2016).

On the other side, multiple sessions tDCS have been successfully used in improving skill acquisition and accuracy, as well task performance. Cantarero et al. (2015) delivered cerebellar tDCS to modulate its activity during a sequential visual isometric pinch task over the course of 3 days. They assessed gains both during training (on-line effects) and between days (off-line effects). They obtained a significant increase in online performance for the participants that received anodal stimulation. Similarly, Benussi et al. (2017) showed a reduction of ataxia symptoms in patients with neurodegenerative cerebellar ataxias following 2 weeks of treatment with anodal tDCS. Therefore, we cannot exclude that with only one tDCS session, the effects may have been minimal, where multiple sessions could have allowed for larger and more long-lasting effects of stimulation to manifest. However, a very recent study has evaluated the effects of three consecutive sessions of anodal cerebellar tDCS on motor learning measured during adaptation to a SBT task and found no significant effect compared to sham stimulation in a healthy population (Kumari et al., 2020). This result speaks against the potential benefit of multiple tDCS sessions on motor adaptation and consolidation. This finding needs to be reproduced in future works.

Although current intensity, density and the time of stimulation was comparable with previous studies, we cannot exclude that cerebellar tDCS would have shown some effect when augmenting the stimulation duration. Moreover, we stimulated the cerebellar hemisphere ipsilateral to the *fast* leg and cannot exclude that cerebellar areas critical for locomotor adaptation might have, at least partly, been spared by the stimulation. There is no clear evidence of laterality regarding cerebellar-dependent adaptation. However, Schlerf et al. (2015) have found stronger connections between the right cerebellum and the left primary motor cortex of right-handed individuals but did not detect lateralized learning processes behaviorally. This points to the importance of modeling for future experiments as age-related differences in skull, differential brain injury and extra-axial space thickness can influence current spread in brain tissue (Bikson et al., 2012). In the same line, our results challenge the polarity assumption (increased/decreased excitability “under” the anode/cathode) of cerebellar tDCS. A recent meta-analysis including 32 sham-controlled studies found no evidence for polarity-dependent effects of cerebellar tDCS (Oldrati and Schutter, 2018) corroborating that the polarity of tDCS might not be predictive of the direction of the behavioral changes in healthy volunteers.

The patients included in the present study were affected mildly to moderately by MS and demonstrated similar learning capacities compared to healthy subjects. Ceiling effects may have been present in this population. Thus, the participants may have performed at maximum possible learning level, preventing further improvement as a result of the stimulation. This may be different in PwMS with higher EDSS scores or in other subjects with neurological diseases where motor learning is known to decline (Dimitrova et al., 2008).

Despite the above-mentioned limitations, we believe that it is important to report negative results. Cerebellar tDCS has been increasingly used in clinical research. Our results clearly underline the urgent need to systematically report both positive and negative results that can help to refine hypotheses for further studies. Because it is difficult to statistically confirm negative findings, they are frequently not published. However, in view of the growing contradictory results and the methodological flaws (Galea et al., 2009; Jalali et al., 2017; Mamlins et al., 2019), it will be of interest to investigate the reproducibility of cerebellar tDCS effects in the future.

## Conclusion

Overall, our study highlights the potential of SBT for adaptation training in PwMS. Transfer and persistence of this ability overground would significantly impact rehabilitation of PwMS. Our results also underline the urge to develop clear references and standards for the application of cerebellar tDCS. To be proposed as a therapeutic option, its effects need to be measurable and replicate reliably in small group designs that reflect the real-world challenges of clinical neurophysiology research and practice. This step is an absolute requirement for the successful application of the technique in clinical routine where a robust effect across behaviors is mandatory.

## Data Availability Statement

The original contributions presented in the study are included in the article/Supplementary Material, further inquiries can be directed to the corresponding author.

## Ethics Statement

The studies involving human participants were reviewed and approved by the Ethics Committee of the Faculty of Medicine, University Hospital of Würzburg, Germany. The patients/participants provided their written informed consent to participate in this study.

## Author Contributions

CN contributed to the conceptualization, investigation, data acquisition, formal analysis, writing – original draft, writing – review and editing. GH contributed to the conceptualization, investigation, data acquisition, and formal analysis. LN contributed to the technical assistance and recruitment. MP contributed to the conceptualization, writing – review and editing. JV contributed to the writing – review and editing, resources, and supervision. DZ contributed to the conceptualization, writing – review and editing, funding acquisition, project administration, resources, and supervision. All authors contributed to the article and approved the submitted version.

## Conflict of Interest

The authors declare that the research was conducted in the absence of any commercial or financial relationships that could be construed as a potential conflict of interest.

## Footnotes

1 http://tarquin.sourceforge.net/
