# Supplementary material for: A Single Session of Anodal Cerebellar Transcranial Direct Current Stimulation Does Not Induce Facilitation of Locomotor Consolidation in Patients With Multiple Sclerosis
Source: Front Hum Neurosci. 2020 Oct 30;14:588671. doi: 10.3389/fnhum.2020.588671 (PMC7661800; doi:10.3389/fnhum.2020.588671)
Supplement: Supplementary file 1 [file Table_1.docx]

**Supplementary table:** Clinical and demographic characteristics of patients with multiple sclerosis (PwMS) and healthy controls (HC). Continuous data are expressed as mean ± standard deviation and ordinal data as median [range].

Abbreviations: F = female; M = male; RRMS = relapsing-remitting multiple sclerosis; SPMS = secondary progressive multiple sclerosis; EDSS = Expanded Disability Status Scale; T25FWT= timed 25 feet walk test; R= right-handed, L = left-handed; MS= multiple sclerosis; DMT = disease-modifying therapy; IF = interferon beta; FN = fingolimod; GA = glatirameracetate; DMF = dimethyl fumarate; FM = fampridine; ALT= alemtuzumab; NAT = natalizumab; OR= ocrelizumab; TR = teriflunomide

| **Experiment 1** |  |  |  |  |  |  |  |  |  |  |  |
| --- | --- | --- | --- | --- | --- | --- | --- | --- | --- | --- | --- |
| **PwMS No** | **Age, y** | **Gender** | **T25FWT(s)** | **Duration of MS, y** | **Disease subtype** | **Current DMT** | **EDSS** | **HC No** | **Age, y** | **Gender** | **T25FWT (s)** |
| 1 | 38 | F | 4 | 21 | RRMS | FN | 1.5 | 1 | 46 | F | 5 |
| 2 | 60 | F | 6.8 | 39 | RRMS to SPMS | GA | 4.5 | 2 | 60 | F | 6 |
| 3 | 58 | F | 6 | 10 | RRMS | DMF | 2.5 | 3 | 58 | F | 4.5 |
| 4 | 41 | M | 5 | 2 | RRMS | DMF | 2 | 4 | 41 | M | 5 |
| 5 | 47 | M | 9 | 21 | RRMS to SPMS | FN | 3.5 | 5 | 51 | M | 4 |
| 6 | 27 | F | 4 | 17 | RRMS | FN | 1.5 | 6 | 32 | F | 3.5 |
| 7 | 56 | F | 5 | 9 | RRMS | FN | 4.5 | 7 | 54 | F | 5 |
| 8 | 56 | F | 4 | 21 | RRMS | FN | 1.5 | 8 | 52 | F | 4 |
| 9 | 54 | M | 6 | 12 | RRMS | FM | 2.5 | 9 | 39 | M | 4 |
| 10 | 40 | F | 7 | 18 | RRMS |  | 2.5 | 10 | 40 | F | 4 |
| **Mean± SD or Median** | **47.8 ± 10.8** |  | **5.7 ± 1.6** | **17 ± 10** |  |  | **2.5**  **[1.5-4.5]** |  | **47.4 ± 8.9** |  | **4.5 ± 0.8** |

| **Experiment 2** |  |  |  |  |  |  |  |  |
| --- | --- | --- | --- | --- | --- | --- | --- | --- |
| ***PwMS sham*** | **Age** | **Gender** | **T25FWT(s)** | **tDCS-mode** | **Duration of MS, y** | **Disease Subtype** | **Current DMT** | **EDSS** |
| 1 | 46 | F | 4.9 | sham | 24 | RRMS | FN | 2.5 |
| 2 | 52 | F | 4 | sham | 5 | RRMS | IF | 2.5 |
| 3 | 43 | F | 3.7 | sham | 5 | RRMS | - | 2 |
| 4 | 41 | F | 4 | sham | 7 | RRMS | IF | 2 |
| 5 | 57 | F | 4.7 | sham | 16 | SPMS | IF | 2.5 |
| 6 | 39 | F | 3.4 | sham | 18 | RRMS | DMF | 2 |
| 7 | 52 | F | 3.8 | sham | 28 | RRMS | FN | 2.5 |
| 8 | 47 | F | 4 | sham | 17 | RRMS | FN | 2 |
| 9 | 28 | M | 4 | sham | 7 | RRMS | DMF | 1.5 |
| 10 | 26 | M | 4 | sham | 5 | RRMS | FN | 1 |
| 11 | 38 | M | 3.1 | sham | 4 | RRMS | FN | 2 |
| 12 | 47 | M | 3.7 | sham | 5 | RRMS | IF | 2 |
| 13 | 34 | M | 4.3 | sham | 8 | RRMS | ALT | 2.5 |
| 14 | 33 | M | 4.1 | sham | 18 | RRMS | IF | 3.5 |
| 15 | 40 | M | 4.8 | sham | 9 | RRMS | GA | 3.5 |
| **Mean±SD**  **or Median** | **41.5 ± 8.9** |  | **4.03 ± 0.5** |  | **13.6 ± 8.2** |  |  | **2[1-3.5]** |
|  |  |  |  |  |  |  |  |  |
| ***PwMS real*** | **Age** | **Gender** | **T25FWT(s)** | **tDCS-mode** | **Duration of MS, y** | **Disease Subtype** | **Current DMT** | **EDSS** |
| 16 | 27 | F | 4.2 | real | 9 | RRMS | FN | 1 |
| 17 | 30 | F | 4.8 | real | 9 | RRMS | FN | 2.5 |
| 18 | 51 | F | 6.05 | real | 16 | RRMS | FN | 3 |
| 19 | 52 | F | 3.2 | real | 20 | RRMS | IF | 2.5 |
| 20 | 54 | F | 6.7 | real | 24 | RRMS | GA | 3 |
| 21 | 34 | F | 4.2 | real | 6 | RRMS | NAT | 1.5 |
| 22 | 52 | F | 4.3 | real | 6 | RRMS | IF | 1.5 |
| 23 | 35 | F | 6 | real | 15 | RRMS | IF | 3.5 |
| 24 | 30 | M | 6.1 | real | 16 | RRMS | FM | 4.5 |
| 25 | 52 | M | 5.4 | real | 14 | SPMS | - | 3.5 |
| 26 | 53 | M | 3.3 | real | 29 | RRMS | - | 2.5 |
| 27 | 49 | M | 4.2 | real | 5 | RRMS | DMF | 1.5 |
| 28 | 53 | M | 4.3 | real | 25 | RRMS | DMF | 3.5 |
| 29 | 24 | M | 4.4 | real | 1 | RRMS | OCR | 2 |
| 30 | 52 | M | 4.9 | real | 9 | RRMS | TR | 2.5 |
| **Mean±SD**  **or Median** | **43.2 ± 11.5** |  | **4.8 ± 1.1** |  | **11.7 ± 7.8** |  |  | **2.5[1-4.5]** |

| ***HC sham*** | **Age,y** | **Gender** | **T25FWT(s)** | **tDCS-mode** | ***HC real*** | **Age,y** | **Gender** | **T25FWT(s)** | **tDCS-mode** |
| --- | --- | --- | --- | --- | --- | --- | --- | --- | --- |
| 1 | 31 | F | 4 | sham | 11 | 27 | F | 5.2 | real |
| 2 | 57 | F | 3.8 | sham | 12 | 57 | F | 4 | real |
| 3 | 48 | F | 5 | sham | 13 | 52 | F | 4 | real |
| 4 | 41 | F | 4 | sham | 14 | 50 | F | 4.5 | real |
| 5 | 35 | F | 4.6 | sham | 15 | 43 | F | 5 | real |
| 6 | 45 | F | 5 | sham | 16 | 26 | F | 3.8 | real |
|  |  |  |  |  | 17 | 39 | F | 5.8 | real |
| 7 | 34 | M | 4.9 | sham | 18 | 35 | M | 3.4 | real |
| 8 | 34 | M | 3 | sham | 19 | 43 | M | 5 | real |
| 9 | 34 | M | 2.5 | sham | 20 | 51 | M | 5 | real |
|  |  |  |  |  | 21 | 56 | M | 5 | real |
| **Mean±SD**  **or Median** | **39.9 ± 8.6** |  | **4.08 ± 0.9** |  |  | **43.5 ± 10.9** |  | **4.6 ± 0.7** |  |
